# The Kuwait National Primary Immunodeficiency Registry 2004–2018

**DOI:** 10.3389/fimmu.2019.01754

**Published:** 2019-07-24

**Authors:** Waleed Al-Herz, Mona Al-Ahmad, Ahmad Al-Khabaz, Ahmed Husain, Ali Sadek, Yasmeen Othman

**Affiliations:** ^1^Department of Pediatrics, Faculty of Medicine, Kuwait University, Kuwait City, Kuwait; ^2^Allergy & Clinical Immunology Unit, Pediatric Department, Al-Sabah Hospital, Kuwait City, Kuwait; ^3^Department of Microbiology, Faculty of Medicine, Kuwait University, Kuwait City, Kuwait; ^4^Department of Allergy, Al-Rashid Allergy Center, Kuwait University, Kuwait City, Kuwait; ^5^Allergy & Clinical Immunology Unit, Pediatric Department, Mubarak Al-Kabeer Hospital, Kuwait University, Jabriya, Kuwait; ^6^Allergy & Clinical Immunology Unit, Pediatric Department, Al-Ameri Hospital, Kuwait City, Kuwait; ^7^Kuwait National Center for Health Information, Ministry of Health, Kuwait City, Kuwait

**Keywords:** immunodeficiency, registry, epidemiology, prevalence, consanguinity, mortality, incidence

## Abstract

**Objective:** To present the report from the Kuwait National Primary Immunodeficiency Registry between 2004 and 2018.

**Methods:** The patients were followed prospectively between January 2004 and December 2018 and their collected data included sociodemographic, diagnosis, clinical presentation, laboratory tests, and treatment.

**Results:** A total of 314 PID patients (165 males and 149 females) were registered during the study period. Most of the patients (*n* = 287, 91.4%) were Kuwaiti nationals and the prevalence among Kuwaitis was 20.27/100,000 with a cumulative incidence of 24.96/100,000 Kuwaitis. The distribution of the patients according to PID categories was as follow: immunodeficiencies affecting cellular and humoral immunity, 100 patients (31.8%); combined immunodeficiencies with associated syndromic features, 68 patients (21.7%); predominantly antibody deficiencies, 56 patients (17.8%); diseases of immune dysregulation, 47 patients (15%); congenital defects of phagocyte number or function, 20 patients (6.4%); autoinflammatory disorders, 1 patient (0.3%); and complement deficiencies, 22 patients (7%). The mean age of the patients at onset of symptoms was 26 months while the mean age at diagnosis was 53 months and the mean delay in diagnosis was 27 months. Most of the patients (*n* = 272, 86%) had onset of symptoms before the age of 5 years. Parental consanguinity rate within the registered patients was 78% and a positive family history of PID was noticed in 50% of the patients. Genetic testing was performed in 69% of the patients with an overall diagnostic yield of 90%. Mutations were identified in 46 different genes and more than 90% of the reported genetic defects were transmitted by an autosomal recessive pattern. Intravenous immunoglobulins and stem cell transplantation were used in 58% and 25% of the patients, respectively. There were 81 deaths (26%) among the registered patients with a mean age of death of 25 months.

**Conclusions:** PID is not infrequent in Kuwait and the reported prevalence is the highest in the literature with increased proportion of more severe forms. Collaborative efforts including introduction of newborn screening should be implemented to diagnose such cases earlier and improve the quality of life and prevent premature deaths.

## Introduction

Primary immunodeficiency diseases (PID) are a genetically heterogeneous group of disorders that affect distinct components of the innate and adaptive immune system predisposing the patients to a wide-range of clinical manifestations including infections, immune dysregulations and malignancies (1). Several national and regional registries were established to study the epidemiology of PID (2–14). They provided an invaluable source of information about the natural history and outcome of specific diseases and documented variabilities between different populations (15–18). They have supported research on the genetic, molecular, and physiological basis of PID (19, 20). They can be used to support clinical trials and translational research to improve quality of care, quality of life, and survival.

Kuwait is a small country in the Arabian Peninsula. In 2018 the population was 4,563,969 [Kuwaiti 1,386,363 (30.3%) and non-Kuwaiti 3,177,606 (69.7%)]^1^. The Kuwait National Primary Immunodeficiency Registry (KNPIDR) was established in 2007 with a set of objectives:

- Determine the prevalence and frequency of different PID in Kuwait
- Identify clinical presentation patterns for PID in Kuwait
- Identify natural history of PID in Kuwait
- Help to assess epidemiology of PID in Kuwait
- Determine particularities about PID affecting the population in Kuwait
- Determine the health impact of PID in Kuwait
- Enhance the knowledge of PID among physicians in Kuwait
- Coordinate clinical research into these disorders
- Development of strategies to improve the care and the quality of life of patients with PID

The calculated prevalence and incidence rates in 2007 in children in Kuwait were 11.98 and 10.06/100,000 respectively (21). In this manuscript we present the report from KNPIDR between 2004 and 2018.

## Materials and Methods

### Data Collection

KNPIDR includes all PID patients diagnosed in Kuwait since 2004. It was approved by The Research and Ethics Committee of the Ministry of Health in Kuwait and the Kuwait University Health Sciences Center Ethical Committee in accordance with the Declaration of Helsinki. An informed written consent was obtained from patients and/or families for whom immunologic or genetic testing was done for research purposes. The patients were followed prospectively between January 2004 and December 2018 and their data was entered into a data form that is divided into 5 sections: sociodemographic data, diagnosis, clinical presentation, laboratory tests, and treatment. The collected data was entered into a computerized database software program which was designed based on the collected data and each patient was given a specific code to ensure confidentiality.

### Patients Diagnosis and Classification

The patients were diagnosed and classified according to the International Union of Immunological Societies, Primary Immunodeficiency Diseases Committee report on Inborn Errors of Immunity (2017) (1). Secondary immunodeficiencies, were ruled out by obtaining detailed history and by performing appropriate testing when suspected. The immunological tests were performed as described previously (22) and included complete blood count with peripheral blood smear evaluation, serum immunoglobulins, antibody response to previous vaccines, lymphocyte phenotyping, and lymphocyte stimulation test. Autoantibodies testing, nitro blue tetrazolium dye test (NBT) or dihydrorhodamine (DHR) and complement hemolytic activity (CH50/100) with specific complement component were done when needed using the standard techniques. Genetic testing was done as described previously (19).

## Results

### Frequency and Distribution of PID

**Figure 1:**
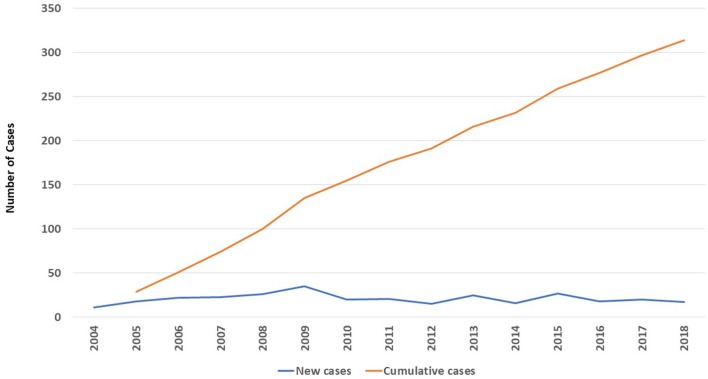
The number (*n* = 314) and annual distribution of registered patients in KNPIDR between 2004 and 2018.

A total of 314 PID patients (165 males and 149 females) were registered in KNPIDR during the study period with an average of 21 cases/year (Figure 1). Most of the patients (*n* = 287, 91.4%) were Kuwaiti nationals, while 27 patients (8.6%) were non-Kuwaitis. The prevalence of PID among Kuwaitis was 20.27/100,000 with a cumulative incidence of 24.96/100,000 Kuwaitis.

**Figure 2:**
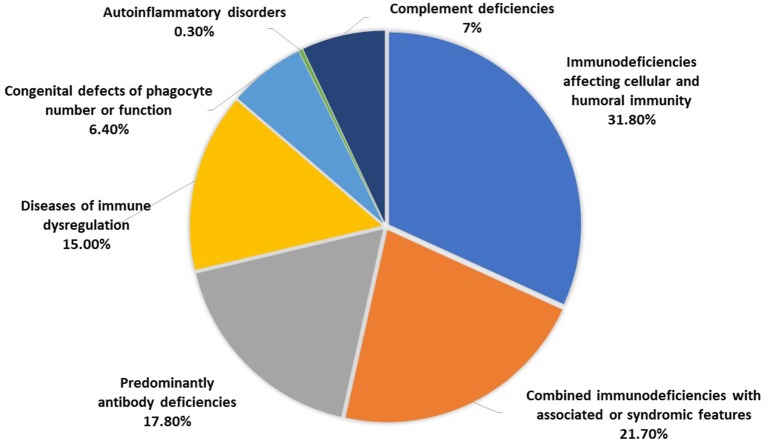
The distribution of patients registered in KNPIDR according to PID categories.

The distribution of the patients according to PID categories was as follows: immunodeficiencies affecting cellular and humoral immunity, 100 patients (31.8%); combined immunodeficiencies with associated syndromic features, 68 patients (21.7%); predominantly antibody deficiencies, 56 patients (17.8%); diseases of immune dysregulation, 47 patients (15%); congenital defects of phagocyte number or function, 20 patients (6.4%); autoinflammatory disorders, 1 patient (0.3%); and complement deficiencies, 22 patients (7%) (Figure 2). No patients with defects in innate immunity were registered. The frequency of each immunodeficiency phenotype in each category is shown in Table 1. Thirteen patients presented as others (4 in the first category, and 4 in the fourth category and 5 in the last category) have PID but could not be categorized according to the IUIS classification system. Of the patients with immunodeficiencies affecting cellular and humoral immunity, MHC II deficiency was the most commonly reported (17 patients) followed by RAG1 deficiency (11 patients), while 22q11.2DS, causing partial DiGeorge syndrome was the most commonly reported among patients with combined immunodeficiencies with associated syndromic features (30 patients). Patients with common variable immunodeficiencies (CVID) were the most common among the predominantly antibody immunodeficiency group (24 patients).

**Table 1:** Frequency of each PID and characteristics of patients from KNPIDR.

**Diagnosis**	**Number of patients**	**Mean onset age**	**Mean delay in diagnosis (Months)**	**Consanguinity**	**Family history**	**IVIG**	**Deaths**
	**M/F**	**(Months)**		**%**	**%**	**%**	**%**
**All patients**	314 165/149	26	27	78	50	58	26
**Immunodeficiencies affecting cellular and humoral immunity**	100 52/48	5	10	95	68	95	44
T-B+	13						
JAK3 deficiency	5						
CD3δ deficiency	3						
T-B-	40						
RAG1 deficiency	11						
RAG2 deficiency	6						
DCLRE1C deficiency	7						
Reticular dysgenesis	5						
ADA deficiency	2						
Combined less profound than SCID	43						
DOCK8 deficiency	10						
DOCK2 deficiency	3						
MHC II deficiency	17						
ZAP70 deficiency	1						
TFRC deficiency	8						
IKBKB deficiency	1						
ICOS deficiency	3						
Others	4						
**Combined immunodeficiencies with associated or syndromic features**	68 35/33	6	27	62	40	23.5	16
PID with congenital thrombocytopenia							
Wiskhott-Aldrich syndrome	3						
DNA Repair Defects							
Ataxia-telangiectasia	11						
ICF	4						
ICF-1	1						
ICF-2	1						
Thymic defect							
22q11.2DS	30						
Immuno-osscous dysplasia							
Cartilage hair hypoplasia	1						
MYSM1 deficiency	5						
Hyper IgE syndrome	8						
STAT3 deficiency	4						
Comel-Nethrton syndrome	1						
Hepatic veno-oclusive disease with immunodeficiency (VODI)	2						
HOIP deficiency	1						
STAT5B deficiency	2						
Immunodeficiency with multiple intestinal atresia	1						
**Predominantly antibody deficiencies**	56 35/21	83	30	52	27	77	3.5
Severe reduction in all serum Ig isotypes with absent B cells	9						
BTK deficiency	6						
μ heavy chain deficiency	1						
Severe reduction in at least 2 serum Ig isotypes with normal or low number of B cells	26						
CVID	24						
NFKB2 deficiency	2						
Severe reduction in serum IgG and IgA with increased IgM and normal number of B cells							
AID deficiency	9						
Isotype, or functional deficiencies with generally normal B cells	12						
Selective IgA deficiency	5						
Transient hypogammaglobulinemia of infancy	7						
**Diseases of immune dysregulation**	47 24/23	20	17	93	55	34	40
Familial hemophagocytic lymphohistiocytosis	21						
Perforin deficiency (FHL2)	4						
Syntaxin 11 deficiency (FHL4)	2						
STXBP2 deficiency (FHL5)	4						
FHL with hypopigmentation	6						
Chediak-Higashi syndrome	4						
Griscelli syndrome	2						
Regulatory T cell defects	4						
FOXP3 deficiency	1						
LRBA deficiency	3						
Autoimmunity with or without lymphoproliferation							
APS-1	3						
Autoimmune lymphoproliferative syndrome	4						
ALPS-FAS	2						
ALPS-FASLG	2						
Immune dysregulation with colitis							
IL10 deficiency	4						
Susceptibility to EBV and lymphoproliferation							
XIAP deficiency	1						
Others	4						
**Congenital defects of phagocyte number or function**	20 9/11	9	13	95	55	25	25
Congenital neutropenia	6						
Glycogen storage disease type 1b	3						
Cyclic neutropenia	2						
JAGN1 deficiency	1						
Defects of mobility							
LAD1	2						
Defects of respiratory burst	9						
CGD p22phox	3						
CGD p47phox	1						
CGD p67phox	1						
Other non-lymphoid defects							
Congenital alveolar proteinosis (CSF2RB)	3						
**Autoinflammatory disorders**	1 M	6	9	100	0	0	0
Blau syndrome	1						
**Complement deficiencies**	22 9/13	71	78	72	54	0	0
C4 deficiency	5						
C8 deficiency	1						
C1 inhibitor deficiency	11						
Others	5						

### Patients Characteristics

**Figure 3:**
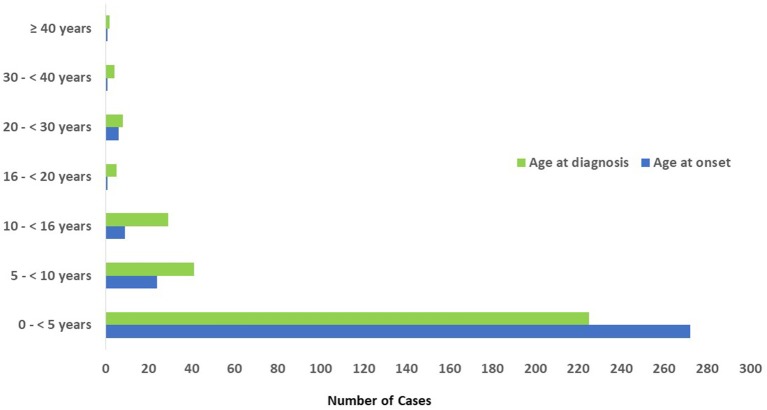
Age distribution of patients registered in KNPIDR according to the onset and diagnosis age.

Adult patients' registration started after the year 2008. The mean age of the patients at the onset of symptoms was 26 months (0 month to 46 years) while the mean age at diagnosis was 53 months (0 month to 53 years). The mean delay in diagnosis, which is defined as the time between the initial presentation and the time of definite diagnosis, was 27 months (0 month to 31 year) (Table 1). Most of the patients (*n* = 272, 86%) had onset of symptoms before the age of 5 year, while 18 patients (5.7%) had onset of symptoms after the age of 10 years (Figure 3). Parental consanguinity rate within the registered patients was 78% with variation among different categories reaching up to 95% in some groups (Table 1). A positive family history of PID was noticed in 50% of the patients, with the highest frequency in patients with immunodeficiencies affecting cellular and humoral immunity (68%).

### Genetic Testing and Management

Genetic testing was performed in 217 patients (69%) with an overall diagnostic yield of 90% (196 patients). Mutations were identified in 46 different genes and more than 90% of the reported genetic defects were transmitted by an autosomal recessive (AR) pattern. Intravenous immunoglobulins (IVIG) were used in 175 patients (58%) while stem cell transplantation was performed in 72 patients (25%), of whom 52 patients belong to the immunodeficiencies affecting cellular and humoral immunity, 10 patients to the immune dysregulation group, 6 to the combined immunodeficiencies with associated syndromic features group, and 4 patients affected by congenital defects of phagocyte number or function.

### Mortality

There were 81 deaths (26%) among the registered patients (Table 1). Forty-four deaths were among patients with immunodeficiencies affecting cellular and humoral immunity. The mean age at death within the overall patients was 25 months (1–40 years). The causes of death were pneumonia, sepsis, multiorgan failure, renal failure, pulmonary hemorrhage, brain hemorrhage, liver failure, severe obstructive cardiomyopathy, and car accident.

### Research Publications

**Figure 4:**
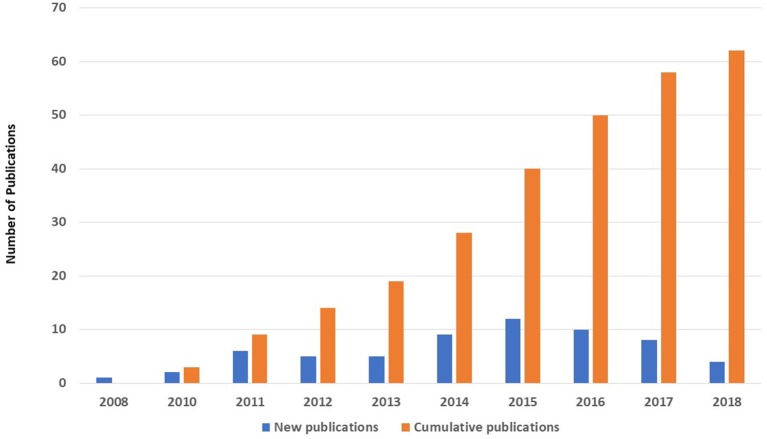
The number (*n* = 62) and annual distribution of original research papers published in MEDLINE with patients' data gathered from KNPIDR between 2008 and 2018.

A MEDLINE (PubMed) search for published papers in the field of PID with patients' data gathered from KNPIDR showed that 62 original research papers were published in peer-reviewed journals during the period from 2008 to 2018 compared to only 1 paper that was published before the establishment of KNPIDR (Figure 4).

## Discussion

Epidemiologic studies are crucial to understanding the burden of chronic diseases on a health care system. In this article, we present the second report from the KNPIDR for the period of 2004–2018.

The prevalence of PID among Kuwaiti nationals is the highest reported in the literature reaching 20.27/100,000 with a cumulative incidence of 24.96/100,000 Kuwaitis. This is compared to lower prevalence rates in the Middle East and North Africa (Qatar 4.3/100,000 children, Morocco 0.81, Oman 7.0 and Tunis 4.3/100,000 inhabitants) (3, 10, 11, 13) and Western countries (United Kingdom at 5.90, France of 8.0, Germany 1.51 and Norway 5.3/100,000) (5, 6, 9, 12). The reported PID prevalence in Iceland was 18.8/100,000 inhabitants (23). This high prevalence was presumed to be due to the easily accessible health care system and that all PID patients are treated in one hospital. Another possible reason is that a central laboratory in the country performs most of all immunological tests. The high prevalence of PID in Kuwait could be explained by many factors such as physicians' awareness, the well-organized referral system to a clinical immunology service, and advances in the country's health system. The high frequency of consanguineous marriages among Kuwaiti population has played an important role in this regard. However, the effect of ethnic and racial variations compared to other populations cannot be eliminated. It is important to mention that the true prevalence is higher than the reported one in this manuscript. This is due to the under-diagnosis of subjects who died before being identified or cases with mild presentation that was missed. Furthermore, patients with autoinflammatory disorders were not registered in KNPIDR since they are being followed by rheumatology services in the country. We have excluded the non-Kuwaiti patients (*n* = 27) from the calculated prevalence since most of them are within the age group of 25–50 years, in contrast to the Kuwaiti population which is, relatively, younger. In addition, most of the non-Kuwaiti are either unmarried or living single in Kuwait while their families are living in their mother countries. Also, most of them stay in Kuwait for short periods (2–5 years) only. We have included the patients with C1 inhibitor deficiency in the prevalence and cumulative incidence calculation like other national registries (5, 8–12, 14).

The registered patients in KNPIDR are characterized by a high proportion of severe forms of PID compared to reports from the Western countries. The very high rate of consanguineous marriages in our registry which exceeded 75% could be the reason for this with a particular increase in the AR diseases that affected more than 90% of patients with known genetic defects. Under-diagnosis of the less severe forms like primary antibody deficiencies may also play a role in these differences. For example, common variable immunodeficiency is the most common PID affecting 30–38% of all PID patients in Germany, Australia, Norway and the United Kingdom (5, 8, 9, 12) compared to only 7.6% in the current report. When compared to the first published report from the KNPIDR in 2007, the proportion of patients with immunodeficiencies affecting cellular and humoral immunity has increased from 21 to 31.8%. This has resulted in an increased proportion of patients treated with HSCT from 10 to 25%. In addition, the deaths have increased from 20 to 26% probably due to the same reason. However, the performance of genetic testing in the current report is substantially higher compared to the first report which is due to the firm international collaboration. This was also reflected on by the number of research publication exceeding 60 original research papers in 10 years. Hence, we believe that the establishment of the KNPIDR has resulted in a systematic interest in PID in Kuwait.

The mean age of the patients at onset of symptoms and diagnosis was 26 and 53 months, respectively, compared to 15 and 43 months in our initial published report. This is probably due to the registration of adult patients which started in 2008. It is important to highlight that only 6% of the registered patients were diagnosed above the age of 16 years. This indicates that attempts should be made to improve internists awareness about PID. Another possible explanation for the low diagnosis of PID in adults is that genetic defects causing CVID may not be prevalent in the Kuwaiti population. The frequencies of parental consanguinity and family history of PID are comparable in both reports.

Consistent with the first report from KNPIDR in 2007, antibody replacement therapy was used in 58% of our registered patients which is comparable to reports from the United Kingdom (60%) and Germany (47%) (5, 12).

Among the important uses of registries is the ability to compare the prevalence of specific disorders between different nations and/or regions. Indeed, we have done this a few years ago by comparing the prevalence of CID between the United States and Kuwait (17). However, we elected not to do this in the current manuscript because of a lack of consistency between different registry reports regarding data collection and, more importantly, in performing genetic studies. In addition, the available national and regional registry reports were published at different time periods. Given the advances in the genetics of PID and our understanding of the pathophysiology of these disorders many such diseases were re-allocated from one category to another, which resulted in more inconsistency between the old reports and the more recent ones.

In conclusion, the KNPIDR continues to provide significant epidemiological data that should advance our understating of the natural history of PID in Kuwait. This must help health authorities to develop plans and to provide the needed resources to improve outcome of PID patients in the country. The prevalence of PID among Kuwaitis is the highest reported in the literature and there is a bias toward more severe forms compared to other regions. Collaborative efforts should be done to early diagnose such cases and improve the quality of life and prevent premature deaths. These include but are not limited to starting a newborn screening program.

## Data Availability

All datasets generated for this study are included in the manuscript/supplementary files.

## Ethics Statement

KNPIDR was approved by The Research and Ethics committee of the Ministry of Health in Kuwait and the Kuwait University Health Sciences Center Ethical Committee in accordance with the Declaration of Helsinki. An informed written consent was obtained from patients and/or families for whom immunologic or genetic testing was done for research purposes.

## Author Contributions

WA-H: establishment and funding of the KNPIDR, patients diagnosis, data collection and analysis, writing the initial manuscript draft, approval of the submitted manuscript, and agreement to be accountable for the content of the work. MA-A, AA-K, and AH: patients diagnosis and data collection, approval of the submitted manuscript, and agreement to be accountable for the content of the work. AS: statistical analysis and approval of the submitted manuscript. YO: data collection and approval of the submitted manuscript, and agreement to be accountable for the content of the work.

### Conflict of Interest Statement

The authors declare that the research was conducted in the absence of any commercial or financial relationships that could be construed as a potential conflict of interest.

## Footnotes

^1^http://stat.paci.gov.kw/englishreports/#DataTabPlace:PieChartNat
